# Imaging Rheumatoid Arthritis in Mice Using Combined Near Infrared and ^19^F Magnetic Resonance Modalities

**DOI:** 10.1038/s41598-019-50043-0

**Published:** 2019-10-04

**Authors:** Hieu Vu-Quang, Mads Sloth Vinding, Maria Jakobsen, Ping Song, Frederik Dagnaes-Hansen, Niels Chr. Nielsen, Jørgen Kjems

**Affiliations:** 1 NTT Hi-Tect institute, Nguyen Tat Thanh University, Ho Chi Minh City, Vietnam; 2 Interdisciplinary Nanoscience Center (iNANO), Aarhus University, Aarhus, Denmark; 3 Center of Functionally Integrative Neuroscience (CFIN), Department of Clinical Medicine, Aarhus University, Aarhus, Denmark; 4 Department of Molecular Biology and Genetics, Aarhus University, Aarhus, Denmark; 5 Department of Biomedicine, Aarhus University, Aarhus, Denmark

**Keywords:** Fluorescence imaging, Magnetic resonance imaging

## Abstract

Rheumatoid arthritis (RA) is an autoimmune disease that causes pain and tissue destruction in people worldwide. An accurate diagnosis is paramount in order to develop an effective treatment plan. This study demonstrates that combining near infrared (NIR) imaging and ^19^F MRI with the injection of labelled nanoparticles provides high diagnostic specificity for RA. The nanoparticles were made from poly(ethylene glycol)-block-poly(lactic-co-glycolic acid) (NP) or PLGA-PEG-Folate (Folate-NP), loaded with perfluorooctyl bromide (PFOB) and indocyanine green (ICG) and evaluated *in vitro* and in a collagen-induced arthritic (CIA) mouse model. The different particles had a similar size and a spherical shape according to dynamic light scattering (DLS) and transmission electron microscopy (TEM). Based on flow cytometry and ^19^F MRI analysis, Folate-NP yielded a higher uptake than NP in activated macrophages *in vitro*. The potential RA-targeting ability of the particles was studied in CIA mice using NIR and ^19^F MRI analysis. Both NP and Folate-NP accumulated in the RA tissues, where they were visible in NIR and ^19^F MRI for up to 24 hours. The presence of folate as a targeting ligand significantly improved the NIR signal from inflamed tissue at the early time point (2 hours), but not at later time points. Overall, these results suggest that our nanoparticles can be applied for combined NIR and ^19^F MRI imaging for improved RA diagnosis.

## Introduction

Rheumatoid arthritis (RA) is a chronic, systemic inflammatory disease affecting approximately 1% of the population worldwide. The disease can lead to the destruction, deformity and functional loss of the affected joints^1^. Being able to establish a diagnosis through imaging could help to better understand RA progression in patients and guide the development of more effective treatment plans.

Macrophages play a central role in RA. An abundance of macrophages is found in the inflamed synovial membrane/fluid and the pannus of inflammatory vascular tissue in RA-affected joints, when compared with healthy controls. Macrophages cause destruction of cartilage and bone in articular joints by producing pro-inflammatory cytokines^2^. During inflammation, activated macrophages also overexpress receptors such as the folate receptor (FOLR1 in human)^3^. Hence, targeting activated macrophages through the systemic administration of nanoparticles is favorable as a diagnostic and therapeutic tool^4,5^ as demonstrated previously using folic acid-functionalised nanoparticles to target activated macrophages in an RA model^6^. However, nanoparticles are quickly removed from circulation by the reticuloendothelial system (RES), thus reducing the number of nanoparticles that accumulate in inflamed joints. It is therefore essential to develop new particles with prolonged half-life and specific targeting to activated macrophages in order to achieve the accumulation in arthritic joints required for diagnostic accuracy and treatment efficacy.

The biopolymers poly(lactic-co-glycolic acid) (PLGA) and poly(ethylene glycol) (PEG) are approved by the Food and Drug Administration (FDA) for systemic administration and their performance in drug delivery is well documented^7^. PLGA has been used as encapsulation material in the vaccination, cancer, inflammation and tissue engineering fields^8^ and can carry many drug types, including hydrophilic and hydrophobic molecules and imaging agents. For example, indocyanine green (ICG), a clinically approved near-infrared (NIR) fluorophore, can be loaded into PLGA particles to help prevent photo-bleaching and systemic clearance and to extend the blood half-life of the fluorophore^9,10^. In addition, PEG is considered a good material for increasing the blood half-life of nanoparticles by preventing the binding between serum plasma and nanoparticles^11^. Thus, the combined use of PLGA and PEG in nanoparticle assembly incorporates all of these advantages^8^. Furthermore, PLGA-PEG can be modified with additional ligands to produce tissue-specific targeting. For example, PLGA-PEG has been conjugated with folic acid to target the overexpressed folate receptors on the surface of certain cancer cells in tumor and activated macrophages in RA joints^3,4,12,13^.

NIR imaging is a reliable diagnostic method. The technique has been applied in pre-clinical trials for various purposes because of its high sensitivity and the rapid generation of images^14^. NIR imaging can be used to study the pharmacokinetics and bio-distribution of nanomedicine *in vivo* by following the fluorescent signal from fluorophore-loaded nanoparticles. For example, Huang Feng *et al*. have developed an RA imaging probe based on fluorescence^15^. However, NIR imaging has disadvantages that make it less applicable for clinical use, such as a lack of anatomical detail in the images, high and unspecific fluorescent background, and low tissue penetration.

In contrast, MRI is a powerful non-invasive medical imaging technique used routinely in the clinic. MRI provides images with high anatomic resolution that are useful for the diagnosis and treatment of many diseases. Certain uses of MRI exploit contrast agents to enhance the specificity of a diagnosis. Among the contrast agents used in clinical and basic research, fluorinated compounds have generated a great deal of attention due to their many advantages. Many fluorinated agents are nontoxic, inert and conveniently monitored using ^19^F MRI. This imaging technique is essentially background-free due to the lack of endogenous ^19^F sources in living systems. In addition, ^19^F MRI enables the direct quantification of fluorine from images, which is useful for evaluating disease states or targeting efficiency. By combining ^19^F MRI and conventional ^1^H MRI it is possible to create highly specific images consisting of ^1^H-based anatomical features and the ^19^F-based detection of the fluorinated compounds.

Perfluorooctyl bromide (PFOB) is one of the perfluorocarbons approved for multiple clinical applications, including acting as a blood substitute because of its inert behavior, biocompatibility and high oxygen solubility, and as an ultrasound contrast agent^16,17^. PFOB can act as a negative contrast agent in gastroenterography, since it does not interfere with ^1^H MRI^18,19^. In addition, PFOB can be used as a positive contrast agent in ^19^F MRI. Similar to most other liquid perfluorocarbons, PFOB exhibits a lipophilicity that prevents its systemic administration. Due to the insolubility of PFOB in water, various nano- and microtechnology methods have been developed to encapsulate PFOB for systemic delivery^20–22^.

Diagnosing RA in patients is currently based on various imaging methods such as MRI and computed tomography^23–27^. Each imaging model provides a distinct set of information for the RA diagnosis such as fast and/or early RA and tissue damage; however, it is necessary to have additional molecular imaging methods to diagnose RA and to quantify the amount of drug in RA therapy. Here, we describe and characterize two novel PFOB- and ICG-loaded nanoparticles composed of PLGA-PEG (NP) or folate-conjugated PLGA-PEG (Folate–NP) and demonstrate their ability to accumulate in activated macrophages in order to diagnose RA in a mouse model by bimodal NIR/^19^F MRI imaging.

## Results

### Nanoparticle characterization

The two types of nanoparticles, NP and Folate-NP, were prepared with the aim to carry a combination of fluorescence and MRI agents specifically to activated macrophages in order to diagnose inflammatory diseases, focusing on RA. The full assembly protocol is described in the materials and methods section. The fluorophore Cou6 was used to track nanoparticle uptake *in vitro* (NP/Cou6 and NP-Folate/Cou6), while NP/ICG and NP-Folate/ICG were used for NIR imaging and MRI *in vivo*. The size, zeta potential and morphology of the nanoparticles were characterized prior to *in vitro* and *in vivo* evaluation. The two types of nanoparticles shared certain similarities. As shown in Table 1, Dynamic Light Scattering (DLS) measurements showed that NP and Folate-NP had the same hydrodynamic size of approximately 150 to 170 nm, a high homogeneity (low polydispersity index (PDI)  < 0.20) and a negative zeta-potential (−42 to −45 mV). The size of the nanoparticles increased to 200 nm in the presence of 10% FBS cell culture medium while the PDI increased to 0.25 due to the adsorption of serum protein on the nanoparticles.

**Table 1:** Hydrodynamic size and zeta potential of NP and Folate-NP. The data are shown as the mean ± SD (*n* = 4).

	Water			10% FBS Culture medium (5 minutes)		10% FBS Culture medium (3 hours)	
	Hydrodynamic size (nm)	PDI	Zeta potential (mV)	Hydrodynamic size (nm)	PDI	Hydrodynamic size (nm)	PDI
NP	171.6 ± 5.99	0.181 ± 0.05	−42.8 ± 2.5	204.2 ± 10.11	0.249 ± 0.05	193.6 ± 11.054	0.240 ± 0.08
Folate-NP	153.7 ± 14.2	0.177 ± 0.04	−45.1 ± 3.2	200.9 ± 7.52	0.231 ± 0.06	193.4 ± 12.992	0.238 ± 0.08

**Figure 1:**
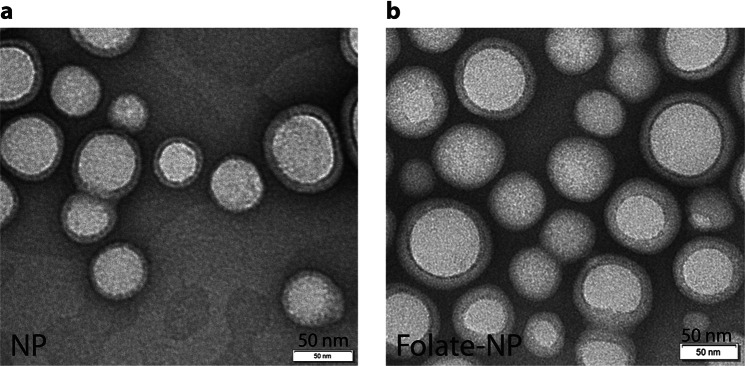
TEM images of NP (**a**) and Folate-NP (**b**). The scale bar corresponds to 50 nm.

The morphology of the nanoparticles, which can influence their blood circulation half-life and drug encapsulation efficiency, was evaluated by TEM. NP and Folate-NP have a spherical shape with a well-defined core-shell structure (Fig. 1). There was no apparent change in the morphology of Folate-NP upon addition of 1% of PLGA-PEG-Folate relative to PLGA-PEG. Aggregation was observed when the weight ratio for PLGA-PEG-Folate in the mixture exceeded 3% (data not shown). The encapsulation efficiency of PFOB and ICG in the nanoparticles was 80% and 0.13%, respectively.

### Folate conjugation increases nanoparticle uptake in macrophages *in vitro*

In order to test for possible cytotoxic effect, we performed MTT cell viability assays for RAW 264.7 macrophages treated with the different nanoparticles. As shown in Supporting Information Fig. S2, Folate-NP and NP only showed minimal cell toxicity (<10% at highest doses (1 mg/ml)) after 24 hrs of treatment. This concentration (1 mg/ml) was used in all subsequent experiments to achieve the highest MRI signal possible.

**Figure 2:**
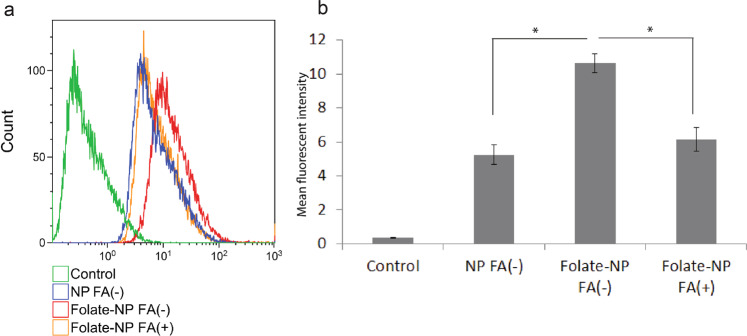
Uptake of nanoparticles in macrophages studied by flow cytrometry. (**a**) Histogram represents the uptake of NP/Coumarin-6 and Folate-NP/Coumarin-6 by Raw 264.7 macrophages with (+) LPS stimulation (1 µg/ml) in the presence (+) or absence (−) of folic acid (FA) (a competitor). (**b**) The mean fluorescent intensity of cells from histogram. The data are shown as the mean ± standard deviation (n = 4), and 10,000 cells were counted. *P < 0.05.

We first studied the uptake of NP and Folate-NP by LPS-activated macrophages *in vitro*. In these experiments, ICG was replaced with Cou-6 as fluorescent tracer in the two nanoparticles (NP/Cou-6 and Folate-NP/Cou-6) to enable detection by flow cytometry. Both particles were taken up efficiently by macrophages (Fig. 2). The mean fluorescent intensity for Folate-NP-treated macrophages in folic-acid-free medium (FA(−); (10.62) was significantly higher than for macrophages treated with NP (5.25), and Folate-NP uptake was specifically inhibited (6.17) in the presence of a competitor (FA(+)) - suggesting a folate receptor-mediated uptake mechanism (Fig. 2). We also observed that there was no significant difference in the uptake of Folate-NP/Cou-6 and NP/Cou-6 in non-activated RAW macrophages by FACS analysis (Supplement Fig. 3). This result agreed with our previous study on the enhanced uptake of folate-labelled particles in a folate-receptor-expressing human epidermal carcinoma cell line (KB)^28^.

**Figure 3:**
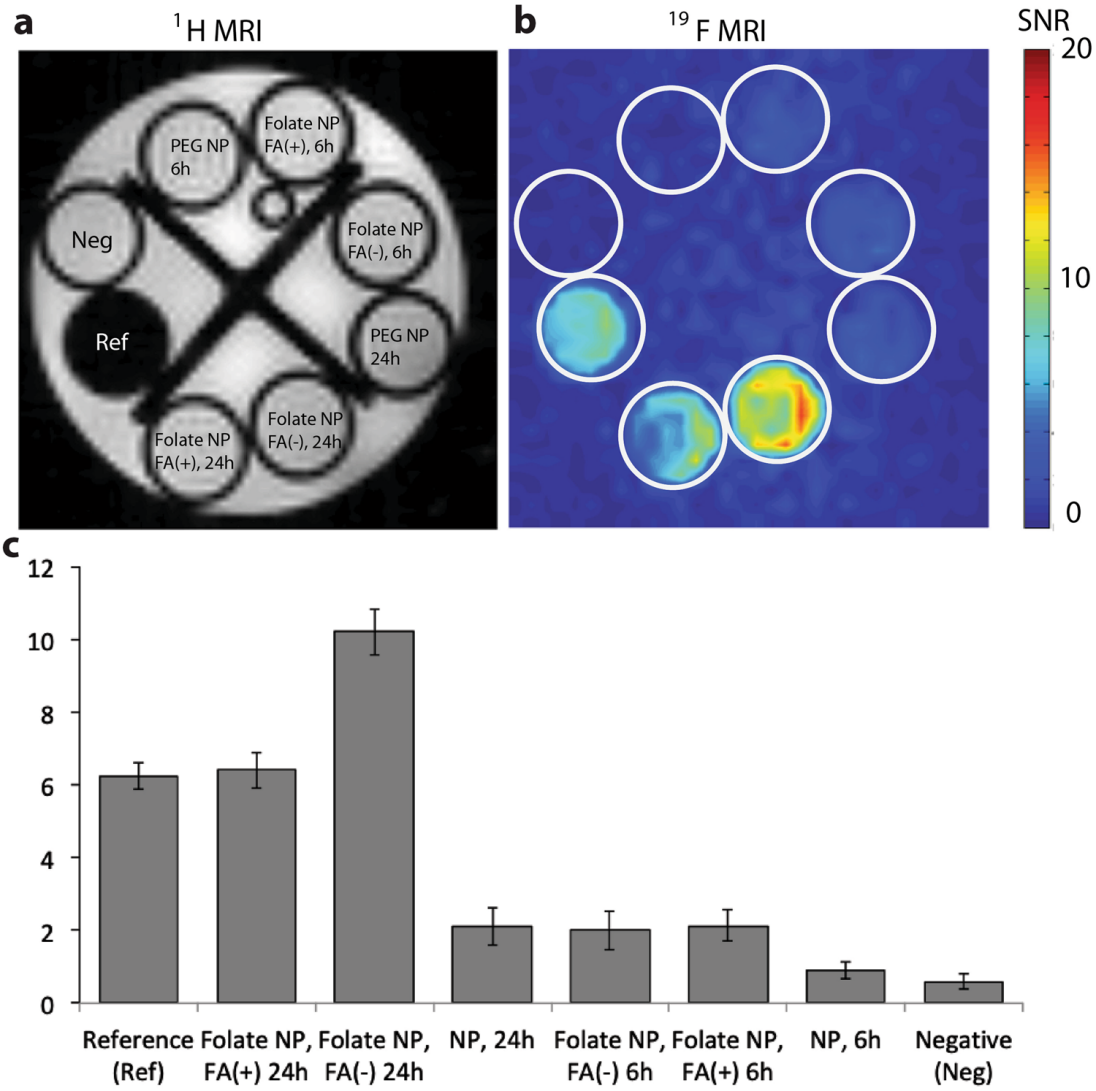
Uptake of nanoparticles in macrophages studied by MRI. (**a**) ^1^H MRI and (**b**) ^19^F MRI of Raw 264.7 macrophages stimulated with LPS (1 µg/ml) in the presence (+) or absence (−) of folic acid (FA) (as competitor) and incubated with NP and Folate-NP (1 mg/ml) for 6 or 24 hours. As reference (Ref) 3.8 µmol of PFOB in CDCl_3_ was used. A total of 2.75 million cells were used. (**c**) Quantification of the ^19^F SNR signal shown in b. Error bars indicate SD, n = 3.

Next, we used ^19^F MRI to study macrophage uptake capacity by evaluating the concentration of fluorinated nanoparticles in the cells. Macrophages are immune cells that phagocytize antigens but it is known that phagocytosis can eventually reach a plateau. Therefore, we quantified macrophage uptake capacity at 6 and 24 hours after addition of the particles, in the presence or absence of FA in the culture medium (Fig. 3). The signal-to-noise ratio (SNR) at 6 hours were 2 and 1.3 for Folate-NP and NP, respectively, and addition of FA in the cell culture medium had no effect on the uptake. The SNR at 24 hours was significantly higher, but only for the Folate-NP particle (10.3 and 6.7 in absence and presence of FA in the culture media, respectively; Fig. 3). This result suggests that uptake saturation did not occur during the first 24 hours of incubation and that activated macrophages mainly engulfed Folate-NP, while only a limited amount of NP was phagocytized.

The signal intensity of the PEGylated NP in RAW 264.7 cells was reduced compared to the non-PEGylated nanoparticles at 2 and 24 hours from ^19^F MRI *in vitro* (Supplemental figure 4). This result implies that the PEG on the nanoparticle surface limits non-specific uptake.

### *In vivo* NIR study in mice with collagen-induced arthritis

In order to test the ability of the nanoparticles to target macrophages in arthritic joints *in vivo*, we used a mouse model for collagen-induced arthritis (CIA). RA was induced in the mice by injecting an emulsion of adjuvant and collagen as previously reported in the literature^29^. Signs of RA started to appear in the feet of the mice 8–12 weeks after induction.

**Figure 4:**
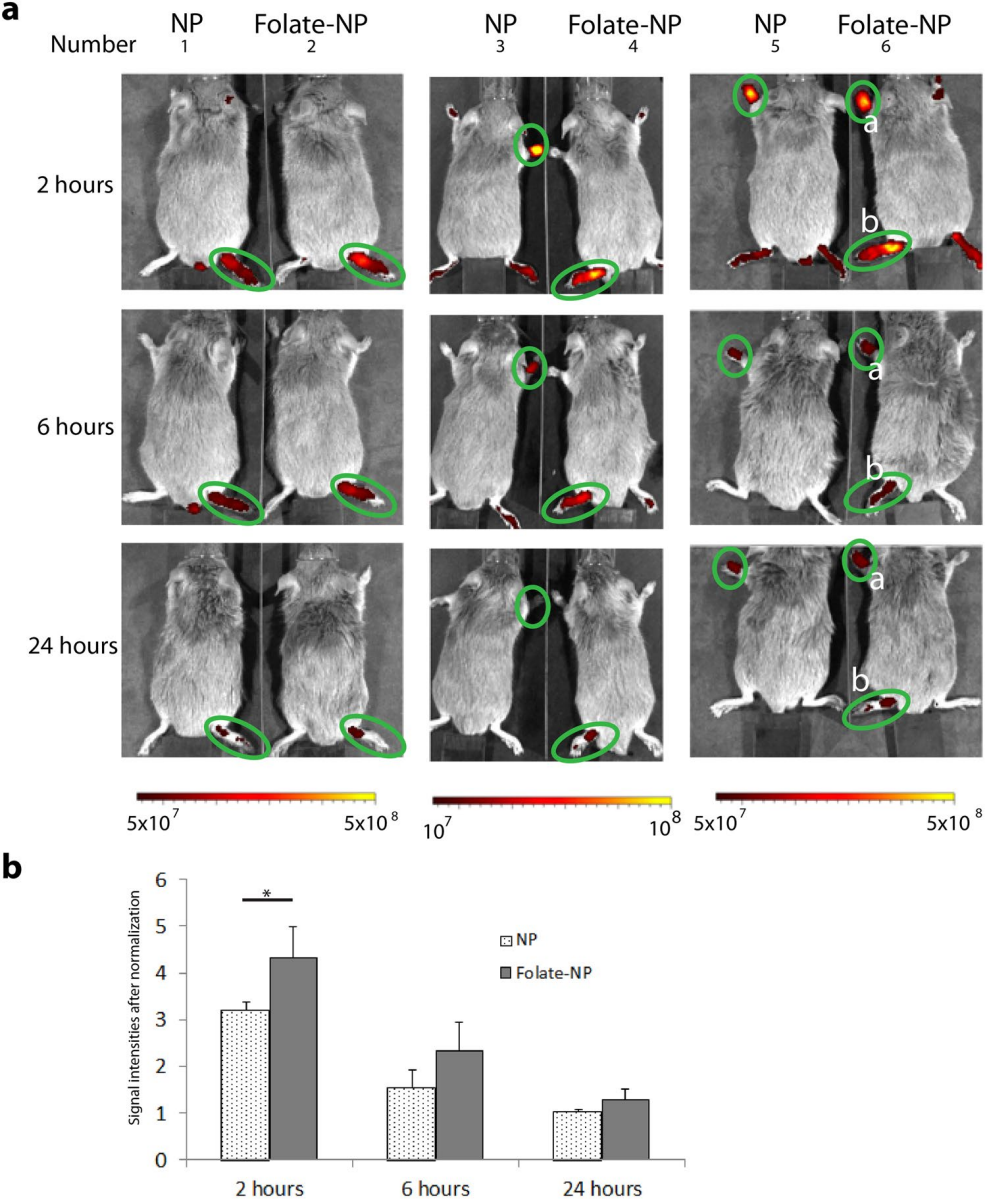
Imaging of RA in mice by NIR detection. (**a**) The near infrared images of the dorsal side of arthritic mice at 2, 6  and 24 hours after an intravenous injection of NP or Folate-NP. (**b**) The quantification of fluorescence signal after normalization within the inflamed foot shown in panel A (green circles). *P < 0.05. Number 6 has two inflamed  paws: front paw No. 6a and hind paw No. 6b (Ventral side is presented in supplemental figure 5).

Mice with similar severity of RA were manually selected, injected intravenously with NP or Folate-NP nanoparticles, and scanned by NIR and MRI at 2, 6 and 24 hours after administration. Untreated or mock-treated mice were included as controls. The NIR imaging showed that both types of nanoparticles accumulate in the arthritic feet and emit fluorescent signals which declined over 2, 6, and 24 hours after injection (Fig. 4). The signal intensities for Folate-NP treated mice after normalization were significantly higher than for the NP treated mice at 2 hours (4.32 ± 0.65 *versus* 3.2 ± 0.16, respectively, P < 0.05). At 6 and 24 hours after injection, signal intensity in the Folate-NP treated animals was slightly higher than in the NP-treated group although this did not reach statistical significance (0.1 > P > 0.05). A fluorescent signal was also found in the peritoneal cavity of mice at 6 hours, although the nanoparticles were injected intravenously, but this was reduced or absent at 24 hours (Supplement Fig. 4).

### *In vivo*^19^F MRI study in mice with collagen-induced arthritis

**Figure 5:**
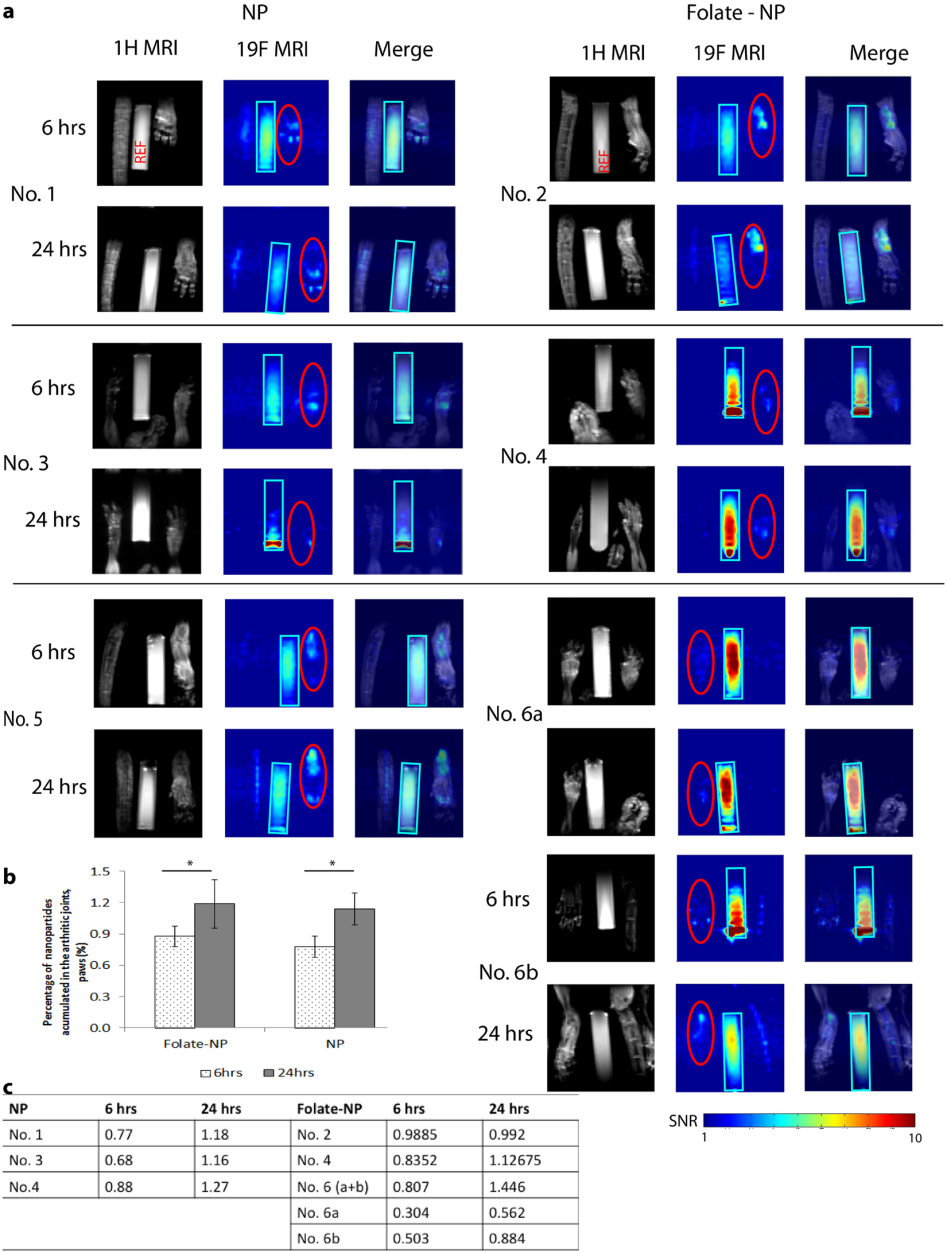
Imaging of RA in mice by MRI detection. The anatomical ^1^H MR image (black/white color code), ^19^F MR image (color code) and image merge at 6 hours and 24 hours post-injection of Folate-NP or NP (**a**). The graph (**b**) represents the accumulation of nanoparticles (%) in the arthritic joints/paws. The accumulations of particle into the inflamed area were the ratios between the ^19^F MRI signals paws (red circle) and REF (light blue rectangular). The table (**c**) presents the percentage of nanoparticles that accumulated into individual arthritic paws and hinds. The data are shown as the mean ± SD (n = 3), *P < 0.05. Number 6 has two inflamed  paws: front paw No. 6a and hind paw No. 6b.

We used ^19^F MRI to evaluate the accumulation of nanoparticles in the arthritic joints (Fig. 5). To better correlate data obtained from the NIR and MRI scans, the mice underwent MRI scanning about 30 mins after the NIR scans. We detected ^19^F MRI signals in the inflamed joints, ankles, whole paws or single toes at 6 and 24 hours after treatment, both with NP and Folate-NP (Fig. 5). Six hours post-injection approximately 0.9 ± 0.1% of the Folate-NP and 0.8 ± 0.1% of the NP injected was only found in the arthritic areas. This increased to 1.19 ± 0.23% and 1.14 ± 0.15%, respectively; at 24 hours and the accumulation of nanoparticles in the RA affected joints could even be seen 48 hours after injection using MRI (data not shown). The isoflurane remaining after anesthesia inhalation during the NIR scan did not give any detectable ^19^F- signal in the MRI scan.

## Discussion

It is desirable to diagnose RA using noninvasive methods in order to improve the treatment and prognosis of the patient. In this study, we set out to investigate whether NIR and ^19^F MRI reporters (ICG and PFOB) encapsulated in NP or Folate-NP could provide such a diagnosis. Both types of nanoparticles were taken up by activated macrophages *in vitro* and in RA tissue *in vivo* confirming their potential as a diagnostic tool. *In vitro*, the activated macrophages took up significantly more Folate-NP than NP, while the two types of nanoparticles showed more similar accumulation in RA affected joints *in vivo*.

PFOB encapsulated in PLGA forms spherical nanoparticles with a well-defined core-to-shell structure. The liquid PFOB is located in the inner core, PLGA/PLGA-PEG forms the shell and PEG is present on the shell surface^20,30^. However, the morphology of the particles and the encapsulation efficiency for PFOB is sensitive to changes in the formulation method. For example, replacing sodium cholate with poly(vinyl alcohol) as a surfactant can change the shape of the particles from a sphere to an acorn^31^. The acorn shape also occurs when PLA-PEG-RGD is added to the formulation recipe^32^. In contrast, we observed no significant change in Folate-NP morphology using our formulation protocol^32,33^. Both Folate-NP and NP showed well-defined core-shell structures (Fig. 1) similar to previous reports^20,30^. This was expected: First, the PLGA used to synthesize PLGA-PEG-Folate had a similar ratio of lactic acid and glycolic acid monomer and solubility  as PLGA-b-PEG. Second, the low concentration of folate (less than 1% weight ratio of PLGA-PEG-Folate) in the particle mixture and its localization at the surface ensures a minimal influence on complexation and size of the particles.

The *in vitro* studies (Figs 2 and 3) determined that NP and, even more pronounced, Folate-NP can target and lead to accumulation inside activated macrophages. The higher accumulation of Folate-NP may be explained by the enhanced binding and endosomal uptake of nanoparticles *via* a specific interaction between folate and its receptor^28,33^. The uptake of NP is presumably due to phagocytosis^4,34^ since both NP and Folate-NP showed similar low fluorescent intensities in non-activated macrophages in FACS (Supplement Fig. 3). Our nanoparticles are produced from biocompatible materials and appeared to be non-toxic to macrophages. This is in line with a previous study where similar nanoparticles were found to be non-toxic to human mesenchymal stem cell, even after being co-incubated for three days^35^.

*In vitro*^19^F MRI experiments showed that in the absence of folate PEGylated NPs were less prone to non-specific uptake than non-PEGylated PLGA PFOB (Supplement Fig. 4). These results clearly showed that PEGylation protects particles from macrophage-mediated phagocytosis in agreement with our previous study^33^. In accordance with this, PEGylated nanoparticles have a longer blood circulation half-life *in vivo* than simpler nanoparticles (t1/2 for NP = 23.9 min *versus* t1/2 for PLGA PFOB = 13.6 min)^20^.

NIR imaging is favored in preclinical trials because it is a fast, multichannel method for obtaining information about nanoparticle targeting, circulation, and association with diseased tissue. In this study, NIR also provided the most significant information. First, NIR could be used to diagnose RA because NP and Folate-NP showed a higher NIR signal in arthritic joints tissues at 2, 6 and 24 hours after administration than in the control animals. Secondly, NIR showed specific targeting of Folate-NP to the arthritic joints compared to NP. In particular, the fluorescent intensity of Folate-NP was higher than NP at 2 hours after injection presumably reflecting specific targeting of Folate-NP/ICG to the folate receptors of activated macrophages in the early time point (Fig. 4). Thirdly, the NIR signals were reduced between 2, 6, and 24 hours post-injection, which may reflect breakdown of the particles in the arthritic joints or metabolism of ICG in the macrophages. Free ICG has a short half-life *in vivo*; therefore, the fluorescence signal will quickly disappear after the release from nanoparticles due to quenching with blood plasma^36,37^. Lastly, the NIR results can also be used to determine the biodistribution of the particles. The nanoparticles were detected in the bloodstream at 2 and 6 hours post-injection, and they were gradually removed 24 hours post-injection (Fig. 4 and Supplement Fig. 5). These data are in agreement with previous reports showing that nanoparticles are eliminated from the bloodstream and accumulate in the large organs such as the liver or spleen after 7 hours^20^.

The *in vivo* ^19^F MRI results supported the NIR analysis by showing accumulation of NP and Folate-NP in the arthritic joint at 6 hours with a significant increase at 24 hours. In agreement with the NIR data, the ^19^F MRI was slightly higher for Folate-NP in the arthritic joint compared to NP; however, these differences did not reach significance. An advantage of MRI is the enhanced anatomical detail about the site of inflammation, for instance, the presence of inflammation in the whole paws, regional joints or a single toe.

The NIR images at 2 hours after nanoparticle administration showed an increased targeting effect of Folate-NP to arthritic joints, however, this target effect was not observed at 6 and 24 hour time points in both NIR and ^19^F MRI. The lack of strong target enhancement using the macrophage targeted Folate-NP at late time points can be explained by three reasons. First, nanoparticles passively accumulate in affected joints simply by being caught in the disrupted capillary, an effect known as the enhanced permeability and retention (EPR) effect, where regional macrophages might phagocytize the nanoparticles independently of the folate receptor^2^. Since there were no difference in size or shape between NP and Folate-NP, their EPR effects are expected to be similar. Secondly, some ligands lose their targeting effects when placed in a biological environment such as blood plasma. A report by Odiu *et al*., 2014 has determined that transferrin (RGD)-functionalized NPs lose their targeting ability *in vivo* because of the absorption of serum protein onto the nanoparticle surface^32,38^. They also reported that there is no significant difference in ^19^F MRI signal in a CT26 tumor upon administration of RGD-NP and NP. The absorption of serum proteins on the nanoparticles could explain the change in particle size (Table 1) that we see in the presence of 10% FBS and this may compromise the targeting of Folate-NP to activated macrophages *in vivo* after long circulation. Another ^19^F MRI report found that the difference between active and passive targeting might be time-dependent^39^. The third reasonable explanation could involve the choice of detection method. NIR is a sensitive molecular imaging method with a wide dynamic range, which may be better used to distinguish between targeting signal and non-targeting signal. However, SNR is one of the limitations of NIR, where the signal is easily affected by many variables such as mouse body and temperature, auto-fluorescence of fur, distance between objects and camera lens, and the low penetration of NIR light into deep tissue. The NIR images were taken on different groups of mice on different days and normalized to the lowest signal in RA at 24 hours, so the standard deviation of the results was high. In contrast to NIR, ^19^F MRI could provide specific diagnosis and quantification since the signal is not affected by depth of tissue. As an example, NIR gave no signal in the liver or spleen at 24 hours (Supplement Fig. 5) while most of the ^19^F MRI signal was found in these organs at this time (Supplement Fig. 6). Thus, the results in ^19^F MRI may be more reliable than NIR. However, ^19^F MRI, and MRI methods in general, provide low sensitivity and was not able to reveal significant differences between targeting and non-targeting nanoparticles in this study. We attribute this to the limited scan time.

Overall, our study demonstrates the potential use of NP and Folate-NP in the diagnosis of RA by combining NIR and ^19^F MRI modalities. Furthermore, this dual imaging technique enables the quantification of nanoparticle accumulation in arthritic joints and the study of drug release kinetics *in vivo*. In the future, our nanoparticle system may also be used to monitor RA treatment or even be loaded with anti-inflammation drugs such as dexamethasone.

## Material and Methods

All reagents were purchased from Sigma-Aldrich, St Louis, MO, USA unless otherwise specified.

### Synthesis of Folate-NP and NP

The NP was formulated as described by Diou *et al*.^20^. The Folate-NP was prepared with the same techniques as NP with a slight modification^33^. In brief, Folate-NP was formulated by dissolving a mixture of PLGA-b-PEG (5050 DLG mPEG 5000, Lakeshore biomaterials, Birmingham, AL, USA) and PLGA-PEG-Folate with weight ratio 100 to 1 (w/w 100/1) and 60 µl of PFOB in 4 ml of dichloromethane. Next, this mixture was emulsified by vortexing (1 minute) and sonicating (1 minute) in 20 ml of 1.5% sodium cholate in an ice bath. The dichloromethane was allowed to evaporate over 4 hours with magnetic stirring. The nanoparticles were collected by centrifugation at 10,000 rpm for 30 minutes and re-dispersed in distilled water. Fluorescent-labeled Folate-NP and NP were produced by adding 1 mg of ICG to the organic solvent before emulsification. For supporting *in vitro* experiments (flow cytometry analysis), we used 50 µg of Coumarin 6 (Cou-6) with a visible fluorescent wavelength instead of ICG. Those nanoparticles are additionally denoted with Cou-6.

PLGA-PEG-Folate were synthesized as described in Vu-Quang et al 2016 and presented in supplementary data^28,33^.

### Measurement of hydrodynamic size

The hydrodynamic size, PDI and zeta potential were measured in deionized water or 10% fetal bovine serum (FBS) medium using a Zetasizer Nano ZS from Malvern Instruments Ltd., Worcestershire, UK. The measurements were completed four times at 25 °C with a scattering angle of 173°.

### Transmission electron microscopy (TEM)

The morphology of the nanoparticles was determined using negative stain TEM. A 10-µl aliquot of nanoparticles was loaded onto a carbon film-coated 200 mesh copper grid (Ted Pella inc., Redding, CA, USA) for 1 minute and then stained with 10 µl of uranyl acetate for an additional 30 seconds. The TEM images were acquired using a TE microscope (Technai G2 Spirit, Oregon, USA) operated at 120 kV.

### Flow cytometry analysis (FACS)

A murine macrophage cell line RAW 264.7 was cultured in folic acid-free RPMI 1640 medium supplemented with 10% FBS and 1% antibiotics at 37 °C and 5% CO_2_ four days prior to the experiment. Cells were harvested and seeded in 24-well plates at a density of 100,000 cells/well. To induce folate receptor expression, 1 µg/ml of liposaccharide (LPS) was added to the media for 24 hours. Next, cells were incubated with Folate-NP/Cou-6 or NP/Cou-6 in the absence (−) or presence (+) (50 ng/ml) of folic acid (FA) in the medium for 3 hours. Cells were washed three times and harvested for the flow cytometry analysis (Beckman Coulter, Pasadena, CA, USA). Four replicates of 10,000 cells each were counted.

### Arthritic *in vivo* model

Animals were housed in type II plastic cages (Techniplast, Italy) in a temperature-controlled pathogen-free animal facility, with unrestricted access to diet (Altromin #1324, Lage, Germany) and tap water. The animal room had a 12:12 hour light-dark cycle (lights on at 06.00). Mice were given nesting material, shredded paper strips, and wood blocks as environmental enrichment. Bedding was aspen wood chips supplied by Tapvei (TAPVEI OY, Finland). The experiments were approved by the Danish Experimental Inspectorate (J. no. 2014-15-0201-00001) and housing of the mice were carried out according to Danish legislation and the Directive 2010/63/on the protection of animals used for scientific purposes. The CIA mouse model was created in DBA/1JRj male mice (7 weeks of age, Janvier Labs, France) using complete Freund’s adjuvant (#7008) and Chicken type II collagen (#20012) and following the guideline from Chronex Inc. (Redmond, WA, USA). Mice with the highest score for one paw and similar conditions were selected for imaging (the scoring rubric is presented in the supporting information)^40^. Due to time limitation in the MRI scans and random RA inflammation on mice’s foot pads, they were numbered and separated into three sub-groups (1 & 2, 3 & 4, and 5 & 6) and experiments were conducted on different days. Number 1, 3, and 5 were intravenously injected with NP, while number 2, 4, and 6 were administrated Folate-NP. Hence, there were three mice in each group. Each mouse was injected intravenously with 200 μl different types of nanoparticle with a PFOB concentration of *C*_PFOB_ = 80 mM. The mice were then scanned side by side using NIR imaging at 2, 6, and 24 hours, and MRI at 6 and 24 hours post-injection. However, in MRI, mice were scanned separately due to the small size of radio frequency coil (25 mm).

### *In vivo* NIR

NIR images of mice under anesthesia with 2.5% isoflurane were obtained using an IVIS® *In Vivo* Imaging System (PerkinElmer, MA, USA) and the fluorescent intensity from the arthritic feet was quantified using the Living Images software version 4.3 (PerkinElmer, MA, USA). An ICG excitation wavelength of 745 nm and 840 nm emission filter was used. The regions of interest were chosen using an automatic operation mode. Then signal intensities were normalized to the lower NIR signal at 24 hours using this formula:

$$NIR\,ratio\,on\,the\,same\,scan\,day=\frac{NIR\,signal\,from\,arthritic\,(at\,any\,times)}{the\,lower\,NIR\,signal\,from\,arthritic\,mouse\,(at\,24\,hours)}$$

### ^19^F MRI

All MRI experiments were performed with a 16.4 Tesla vertical bore Bruker Advance III spectrometer (Bruker BioSpin, Rheinstetten, Germany) running the ParaVision 6.0 software. Data were analyzed with MATLAB (MathWorks, Natick, MA, USA).

#### *In vitro*^19^F MRI

Folate receptor-activated Raw 264.7 cells were incubated with Folate-NP or NP under various conditions. Next, cells were washed three times and harvested. Tubes of 2.75 × 10^6^ cells were placed in each tube to obtain the ^19^F MRI images. The multi-slice multi-echo (MSME) pulse sequence was used with an echo time (TE) of 3.36 ms, a repetition time (TR) of 5 s, a field of view (FOV) of 22 × 22 mm^2^, an image matrix of 32 × 32, 23 averaging scans (NA), and an excitation frequency of −83 ppm. The chemical shift artifacts of PFOB were avoided by following the concepts introduced in ref.^41^ with pulse bandwidths of 4.5 kHz. The total duration of the *in vitro* MRI scans (including anatomical ^1^H MRI scans) was approximately one hour. The ^19^F MRI signal of the cells in the tubes was measured three times.

#### *In vivo* MRI

The mice that underwent NIR scans were anesthetized by an intra-peritoneal injection of 200 µl of a ketamine/xylazine/PBS (90 mg/kg K, 10 mg/kg X) mixture prior to the MRI scans. The mice were placed in the imaging probe with an ambient temperature of 28 °C. The ^19^F MRI was obtained using the MSME sequence with a FOV of 25 × 25 mm^2^, image matrix size of 32 × 32, 30 equidistant spin echoes with a TE ranging from 2.42 ms to 72.6 ms, a 5 s TR, an NA of 12, and a 20 mm slice thickness. For figure presentation, the first echo images were chosen and posted- processed using Matlab (2012). The images were resized to [1024 1024], with “bi-linear” function, and color map “jet”. The ^1^H MRI was obtained using the MSME sequence with a FOV of 25 × 25 mm^2^, image matrix size of 64 × 64, TE 7 ms, a 5 s TR, an NA of 12, and a 0.5 mm slice thickness, number of slice 7.

The total duration of the *in vivo* MRI scans (including anatomical ^1^H MRI scans) was approximately one hour for each mouse. Our ^19^F MRI method was not sensitive to the previous isoflurane inhalation remaining  after NIR scans.

The ^19^F signal-to-noise ratio (SNR) maps were obtained by taking the magnitude ^19^F signal from 1^st^ echo image and dividing by the noise. Noise intensity was taken from the corner of the images. The accumulation-percentages of nanoparticles in the arthritic joints were calculated by comparing the ^19^F signal from the arthritic joints with the signal from a reference sample (with concentrations of nanoparticles equal to 1/30 of the injection dose) placed in a glass tube in the vicinity of the arthritic joint. To eliminate the influence of noise in the analysis, pixels with intensities below 1.4 were masked out.

Statistically, the student t-test was used to calculate the probability (*P*) distribution in the study, with two samples assuming unequal variances, two tails distribution.

## Supplementary information

Supporting information
