# Supplementary material for: Imaging Rheumatoid Arthritis in Mice Using Combined Near Infrared and 19F Magnetic Resonance Modalities
Source: Sci Rep. 2019 Oct 4;9:14314. doi: 10.1038/s41598-019-50043-0 (PMC6778085; doi:10.1038/s41598-019-50043-0)
Supplement: Supplementary file 1 — Supporting information [file 41598_2019_50043_MOESM1_ESM.pdf]

Imaging Rheumatoid Arthritis in Mice Using Combined Near Infrared and ^{19}F Magnetic Resonance Modalities

Hieu Vu-Quang[1,2], Mads Sloth Vinding[3], Maria Jakobsen[4], Ping Song[2], Frederik Dagnaes-Hansen[5], Niels Christian Nielsen[2], Jørgen Kjems[2,4*]

1 NTT Hi-Tect institute, Nguyen Tat Thanh University, Vietnam

2 Interdisciplinary Nanoscience Center (iNANO), Aarhus University, Denmark

3 CFIN center, Department of Clinical Medicine, Aarhus University, Denmark

4 Department of Molecular Biology and Genetics, Aarhus University, Denmark

5 Department of Biomedicine, Aarhus University, Denmark

Correspondence:

Professor Jørgen Kjems

Interdisciplinary Nanoscience Center (iNANO)

Aarhus University

Gustav Wieds Vej 14

DK-8000 Aarhus

Denmark Tel + 45 28992086 Email jk@mbg.au.dk

Supporting information

PLGA-PEG-Folate synthesis

The PLGA-PEG-Folate polymer was synthesized as described by Esmaeili et. al, 2008 with minor modifications[1]. Briefly, the carboxylic group of PLGA resomer RG 504H (Evonik industries, Germay) was converted into PLGA-NHS using N-hydroxysucconimide (EDC)/1-ethyl-3-(3-dimethylamino) carbodimide (NHS) with molar ratio 1:10:10 for 24 h in dry DCM. The polymer was precipitated in ice-cold methanol, centrifuged and washed 3 times to remove unreacted agent and dried using vacuum. The PLGA-NHS was then reacted with bis-amine 6 kDa PEG with molar ratio (1:5) in dry DCM for a 24 hours. The reaction was precipitated in a cold mixture of methanol and diethyl ether, centrifuged and washed 3 times and dried under low pressure. Folic acid (13 mg) was activated with N,N'-dicyclohexylcarbodiimide DCC (13 mg) in dry Dimethyl sulfoxide (DMSO) overnight. Next, PLGA-PEG-NH_2 was added to react with folic acid overnight and slowly dropped into 50 ml mixture of methanol/water before transfer to a 14 kDa dialysis membrane to remove unconjugated product for 2 days (water were changed twice a day). The final product was freeze dried and kept at -20°C. The conjugation of folic acid to PLGA-PEG was confirmed by NMR Bruker 500 MHz (Bruker, Billeria, MA, USA). The concentration of folic acid in the polymer was optimized using UV-Vis at wavelength (280 nm to 285 nm)

Weight of nanoparticles

One hundred microliters of purified nanoparticles (n =3) were put in an Eppendorf tube and freeze dried for 6 hours. The net weights of nanoparticles were measured using the scale.

Measuring concentration of PFOB and ICG loaded nanoparticle

Concentration of PFOB in the nanoparticles was estimated using ^{19}F MRI. In brief, 100 µl of purified nanoparticles were placed in an NMR tube together with various concentration of PFOB in $CDCl_3$. The multi-slice multi-echo MSME pulse sequence was used with an echo time (TE) of 3.36 ms, a repetition time (TR) of 12 s, a field of view (FOV) of 22x22 mm^2, slice thickness 10 mm, an

image matrix of 32x32, 10 averaging scans (NA), and an excitation frequency of -83 ppm. After the MRI acquisition, the ^{19}F SNRs of each NMR tube were measured and standard curves were built. The amounts of PFOB in nanoparticles were then calculated using the standard curve.

The encapsulation efficiency of PFOB in nanoparticle was calculated by comparing the ratio of amount PFOB/weight of nanoparticle before formulation and after purification.

The concentration of ICG in nanoparticles was measured by DMSO extraction. Briefly, ICG-loaded nanoparticles (after freeze drying) were dissolved in 500 µl of DMSO and shaken for 3 h. The solution was then centrifuged at 17,000 g for 10 min, to remove all precipitants. The concentration of ICG was measured using a Fluorometer (Horiba-Yvon, Japan) at an excitation wavelength of 765 nm and the concentration was calculated using a standard curve from a various ICG solutions.

Cell viability assay

Experiments were performed in 96-well plates where RAW macrophages 264.7 (10^4 cells/well) were treated with various concentrations of Folate-NP and NP at (0, 0.25, 0.5, 0.75, 1 mg/ml, n =4). After 8 hours of incubation, the media was renewed and cells were grown overnight. On the next day, a standard MTT assay was performed according to the manufacturer's protocol (Sigma) in order to estimate cell viability. Data was subject to the student t-test and $P<0.05$ was considered statistically significant.

Rheumatoid arthritis scoring

RA developed in mice at 4 to 8 weeks after induction. The following rubric score was used to access the extent of the RA:

0 No evidence of erythema and swelling

1 Erythema and mild swelling confined to the tarsals or ankle joint

2 Erythema and mild swelling extending from the ankle to tarsals

3 Erythema and moderate swelling extending from the ankle to metatarsal joints

4 Erythema and severe swelling encompassing the ankle, foot and digits or ankylosis of the limb

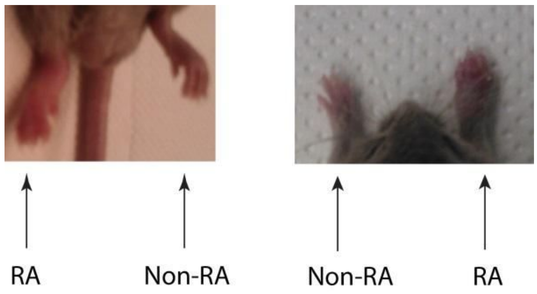

Representative images of mice with RA that were selected for treatment.

Supplementary data

Characterization

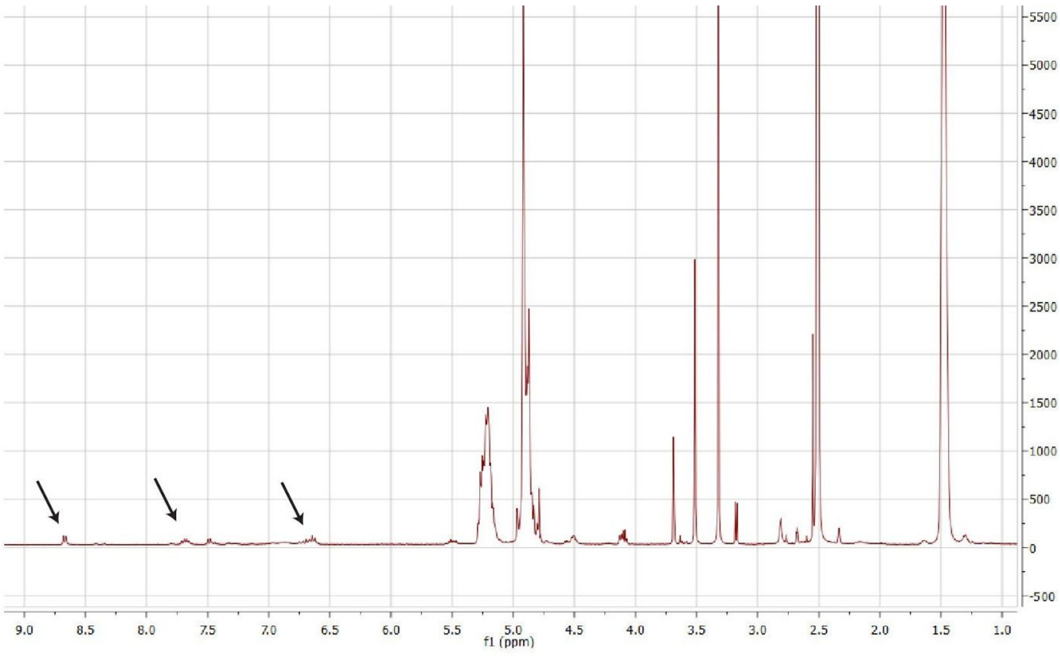

Supplement Figure 1: ^{1}H NMR spectrum PLGA-PEG-Folate in d6-DMSO. The encircled small peaks at position -8.68, -7.67, -6.65 ppm confirm that folic acid has been conjugated to PLGA-PEG.

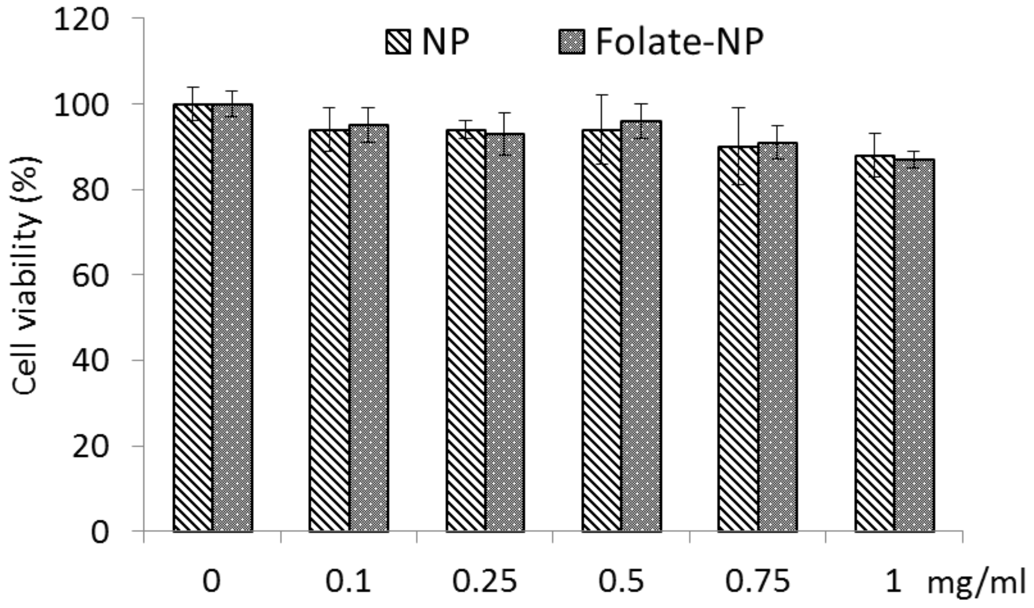

Supplement Figure 2: Cell viability. Standard MTT assays were performed on Raw 264.7 macrophage to measure cell viability after nanoparticle incubation. Cells were incubated for 24 hrs with NP and Folate-NP at concentrations 0, 0.1, 0.25, 0.5, 0.75, 1 mg/ml. The data are presented as the mean ± SD, *n* = 4.

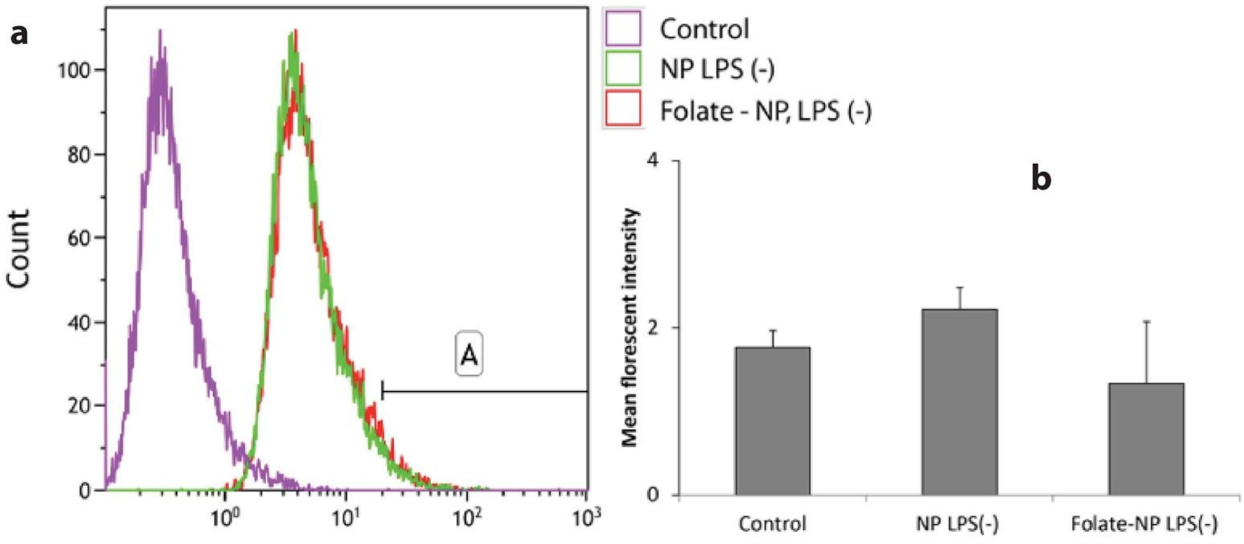

Supplement figure 3: (a): histogram represents the uptake of NP/Coumarin-6 and Folate-NP/Coumarin-6 by Raw 264.7 macrophages without LPS stimulation (1 µg/ml). (b) Mean

fluorescent intensity of cells from histogram shown in panel a. The data are shown as the mean ± standard deviation (n = 4). 10,000 cells were counted. * $P<0.05$.

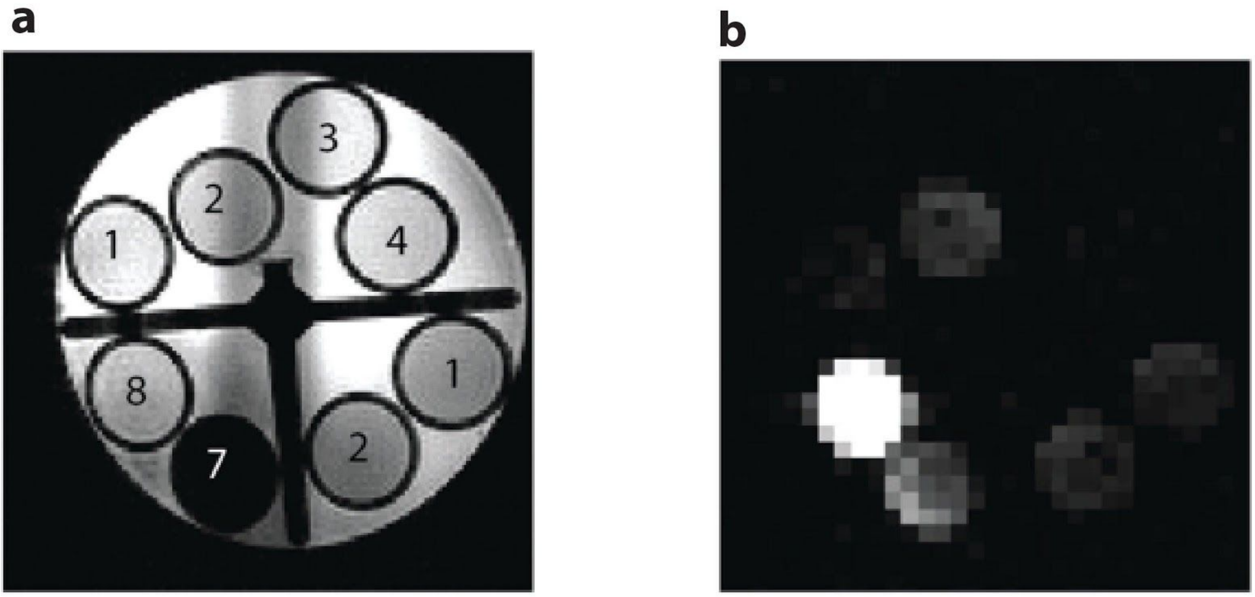

Supplement figure 4: Magnetic resonance imaging of un-activated Raw 264.7 macrophages incubated with NP and PLGA-PFOB (nanoparticle without PEG). (a): ^{1}H MRI signals, (b): ^{19}F MRI signals, indicating the arrangement of tubes. Tube 1: Raw 246.7 & NP 24hrs, tube (mean SNR 7.675 ± 0.9) 2: Raw 246.7 & fluorinated nanoparticle without PEG 24 hrs. (mean SNR 5.22 ± 1.01), tube 3: Raw 246.7 & Raw 246.7 at 2 hrs., tube 4: fluorinated nanoparticle without PEG at 2 hrs, tube 7: $3.8x10^{-3}$ mmol of PFOB in $CDCL_3$ tube 8:NP in water. Number of cells: 10^6 cells.

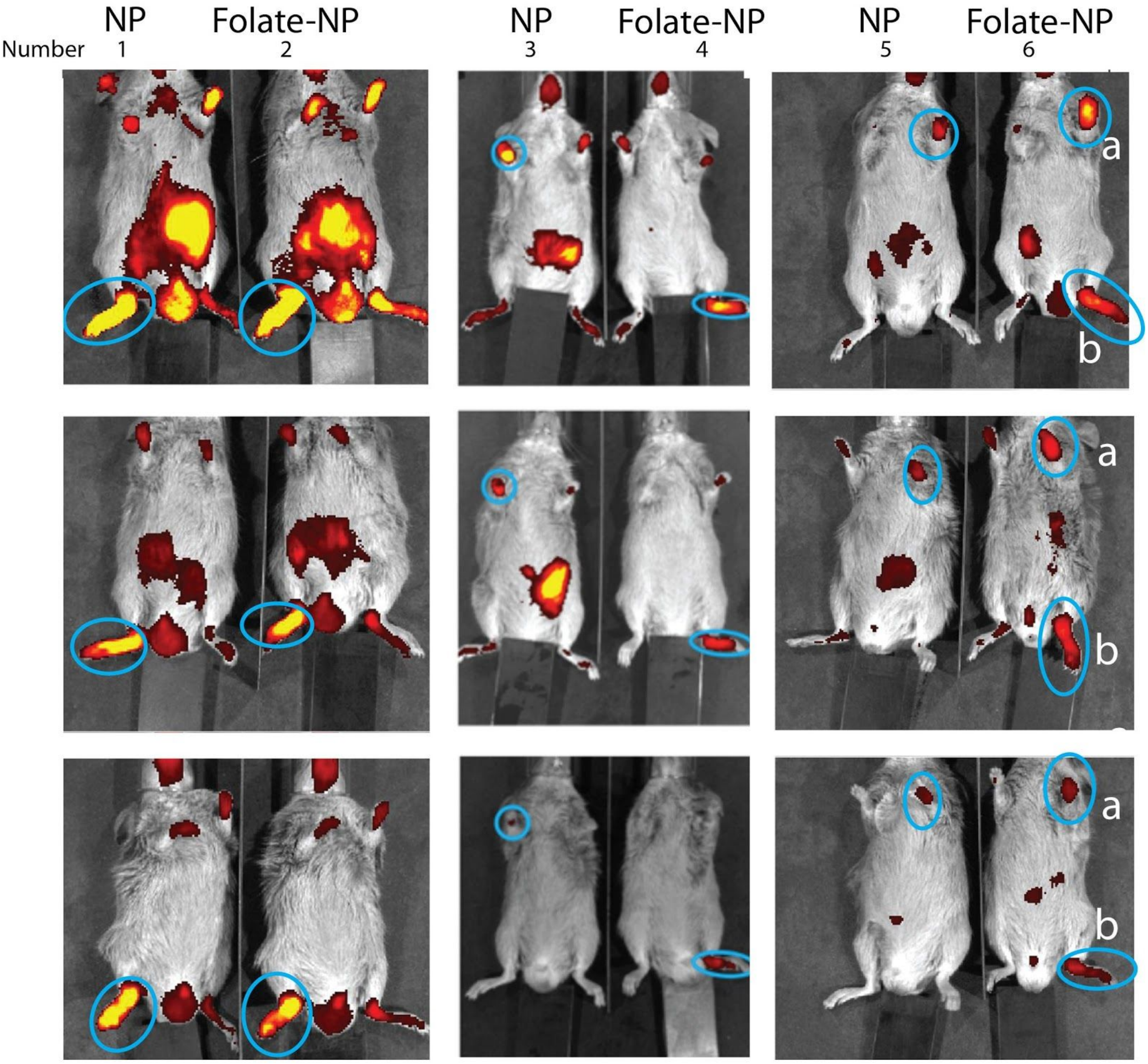

Supplemental Figure 5: NIR images of the ventral sides of arthritic mice at 2, 6 and 24 hours after I.V. injections of NP/ICG or Folate-NP/ICG. The inflamed joints are indicated by blue circles. Mouse #6 has two inflamed paws: front paw (a) and hind paw (b).

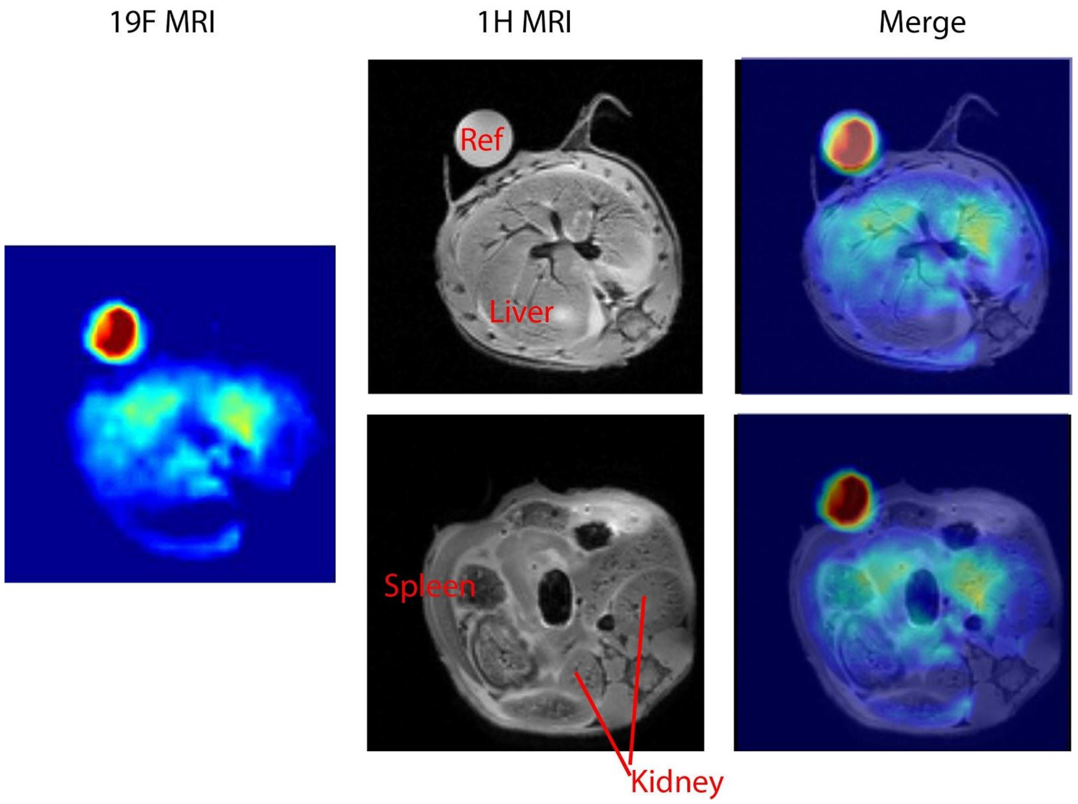

Supplemental Figure 6: Mouse body scan made 24 hrs after injection of NP. The ^{19}F MRI shows accumulation of the NPs in the abdominal area. ^{1}H MRI depicts the anatomical details of the mouse, showing that most of the NP accumulates in the liver and spleen.

1 Esmaeili, F. *et al.* Folate-receptor-targeted delivery of docetaxel nanoparticles prepared by PLGA–PEG–folate conjugate. *Journal of Drug Targeting* **16**, 415-423, doi:10.1080/10611860802088630 (2008).
